# Impact of anatomical versus IDEAL anterior cruciate ligament reconstruction on postoperative functional recovery

**DOI:** 10.1097/MD.0000000000049590

**Published:** 2026-07-10

**Authors:** Jianbin Gu, Xiaoqi Zhou, Yanpeng Fu, Xun Zhen, Zhizhu Xu, Zhaoyu Gong

**Affiliations:** a Department of Joint Sports Medicine, People’s Hospital of Jilin City, Jilin, Jilin Province, China.

**Keywords:** anatomical reconstruction, anterior cruciate ligament (ACL) reconstruction, IDEAL reconstruction, postoperative functional recovery

## Abstract

Anterior cruciate ligament (ACL) tears are common injuries that often require reconstruction to restore knee stability and function. Anatomical reconstruction (AR) seeks to replicate the native ACL footprint and orientation, whereas isometric, direct insertion, eccentrically located, anatomical, low tension (IDEAL) reconstruction applies principles intended to optimize graft positioning and tension behavior across knee motion. This retrospective study compared postoperative functional recovery between AR and IDEAL techniques in patients treated at our institution from January 2020 to December 2021. A total of 76 patients with confirmed ACL tears were included: 39 patients underwent individualized AR and 37 received IDEAL reconstruction. Functional outcomes were assessed using the Lysholm Knee Scoring Scale and the International Knee Documentation Committee (IKDC) subjective score at 3, 12, and 24 months postoperatively. Analyses were performed using SPSS, with statistical significance defined as *P* < .05. Both groups demonstrated continuous improvements in Lysholm and IKDC scores across follow-up. At each postoperative time point (3, 12, and 24 months), there were no statistically significant differences between the AR and IDEAL groups for either Lysholm or IKDC scores (all *P* > .05). These findings indicate similar functional recovery patterns for both reconstruction strategies within the observed period. In summary, AR and IDEAL reconstruction were associated with substantial postoperative functional gains and showed comparable patient-reported outcomes through 24 months. Given the retrospective design and limited sample size, larger prospective randomized studies are required to confirm equivalence, clarify patient selection criteria, and assess longer-term durability and safety.

## 1. Introduction

Anterior cruciate ligament (ACL) tears are among the most common and debilitating injuries in orthopedics, predominantly affecting athletes and individuals participating in high-intensity physical activities. The ACL serves as a critical stabilizer of the knee joint, preventing anterior translation and controlling rotational stability of the tibia relative to the femur.^[1–3]^ Rupture of the ACL frequently results in substantial functional impairment, joint instability, and an increased risk of secondary injuries, including meniscal tears and post-traumatic osteoarthritis. Consequently, effective surgical reconstruction is essential for restoring knee stability and function, thereby enabling patients to return to their pre-injury levels of activity.^[4,5]^

Over the past few decades, ACL reconstruction techniques have undergone substantial evolution, with AR widely regarded as the gold standard. This approach aims to restore the native ACL anatomy by precisely replicating its original femoral and tibial insertion sites. Typically, bone tunnels are created at the anatomical footprints of the torn ligament to re-establish physiological knee kinematics postoperatively. Anatomical reconstruction (AR) has been associated with improved graft incorporation, enhanced rotational stability, and higher rates of return to pre-injury activity levels compared with nonanatomical techniques.^[6–8]^ Despite its widespread adoption, emerging evidence has identified potential advantages of the isometric, direct insertion, eccentrically located, anatomical, low tension (IDEAL) reconstruction technique. The IDEAL approach integrates several key principles designed to optimize surgical outcomes. Isometric positioning maintains consistent graft length throughout the range of motion, reducing the risk of elongation and failure. Direct insertion ensures accurate placement of the graft at the ACL’s native insertion sites to maximize biomechanical function.^[9,10]^ Eccentric location involves positioning the femoral tunnel in the anterior (high) and proximal (deep) regions of the ACL footprint to enhance stability. Anatomical placement ensures the graft lies entirely within the native footprint, while low tension minimizes excessive graft stress during the healing process.^[11,12]^

Recent investigations have compared ACL graft positioning strategies, revealing that AR better restores native knee kinematics than isometric/IDEAL placements, which may induce increased anterior tibial translation and internal rotation.^[13]^ Clinical studies indicate that variations in graft isometry within AR do not significantly affect short-term outcomes, underscoring the complexity of optimal tunnel placement.^[14]^ Imaging-based research has defined precise tunnel characteristics via anteromedial portal techniques, highlighting the need for individualized tunnel orientation.^[15]^ In addition, computational models have delineated femoral isometric zones and identified key anatomical landmarks for synthetic graft placement.^[16]^ However, few studies have directly compared IDEAL reconstruction, which integrates isometric, direct insertion, and low-tension principles, with individualized AR in clinical practice. This study aimed to address this gap by comparing the functional recovery outcomes of both techniques in a real-world cohort, thereby providing new insights to guide surgical decision-making based on both biomechanical rationale and clinical effectiveness.

## 2. Methods

### 2.1. Study design

This study was approved by the Ethics Committee of People’s Hospital of Jilin City. A retrospective evaluation was conducted at our institution to compare the impact of AR and IDEAL reconstruction on postoperative functional recovery in patients with ACL tears. Patients were recruited consecutively based on the date of surgery; all eligible individuals underwent ACL reconstruction between January 2020 and December 2021 and were followed through December 2023 to allow for a minimum of 24 months of postoperative assessment. To ensure data completeness, only patients with full preoperative and postoperative evaluations were included; consequently, all 76 enrolled patients contributed follow-up data at each scheduled time point. The research methodology, intent, and protocols were meticulously designed in accordance with the Strengthening the Reporting of Observational Studies in Epidemiology guidelines.^[17]^ This study was subsequently approved by the Ethics Committee of our hospital.

### 2.2. Inclusion and exclusion criteria

Inclusion criteria:

1. Age range: patients aged 18–50 years.
2. Diagnosis: confirmed ACL tear diagnosed via magnetic resonance imaging (MRI) and clinical examination.
3. Surgical intervention: patients who underwent either AR or IDEAL reconstruction of the ACL within the specified study period (January 2020–December 2021).
4. Follow-up: availability of complete preoperative and postoperative data, including clinical assessments and patient-reported outcome measures.

Exclusion criteria:

1. Multiligament injuries: patients with concomitant injuries to other knee ligaments (e.g., posterior cruciate ligament, medial collateral ligament, and lateral collateral ligament).
2. Previous knee surgeries: patients with a history of previous surgical intervention on the affected knee.
3. Significant comorbidities: patients with comorbid conditions that could affect postoperative recovery, such as severe osteoarthritis, rheumatoid arthritis, or other systemic inflammatory conditions.
4. Additional knee pathologies: the presence of other significant knee pathologies, such as extensive meniscal tears requiring repair, chondral lesions, or other structural abnormalities.

### 2.3. Surgical techniques for anatomical and IDEAL reconstruction

All surgical procedures were performed by experienced orthopedic surgeons from the same sports medicine team, each of whom had substantial prior experience in ACL reconstruction techniques before the initiation of the study period. Both AR and IDEAL reconstruction procedures were routinely performed during the same time interval rather than being sequentially adopted techniques, thereby reducing the potential influence of temporal bias and learning-curve effects.

#### 2.3.1. General surgical procedure

All surgeries were conducted under general anesthesia. A standard anterolateral and anteromedial portal was established to access the knee joint. The joint cavity was thoroughly inspected, and a complete ACL tear was confirmed. In cases where meniscal injuries were present, these were addressed first through repair or partial meniscectomy. The ACL remnants were then debrided to prepare for reconstruction. The semitendinosus tendon from the ipsilateral leg was harvested for ACL reconstruction. After removing any attached muscle tissue, the tendon was braided at both ends using No. 2 Ethibond nonabsorbable sutures and folded to create a quadruple-strand graft. The diameter of the graft was measured with a precision of 0.5 mm. Femoral and tibial tunnels were created with diameters matching the graft. The graft was then introduced through these tunnels. Femoral fixation was achieved using an Endobutton, while tibial fixation was accomplished with an interference screw, ensuring proper tension with the knee in full extension. The surgical wound was closed, and the knee was immobilized in full extension with a brace.

#### 2.3.2. AR group

For patients in the AR group, the surgical approach followed the individualized AR protocol as proposed by Professor Freddie. Preoperative MRI was utilized to measure the size of the femoral ACL remnant, which informed the selection of an appropriately sized graft. The cross-sectional area of the graft was designed to cover 50% to 80% of the tibial footprint area. Using an anteromedial portal for precision, the femoral tunnel was positioned at the anatomical footprint of the ACL or based on the center of the remnant. This method aimed to closely replicate the natural anatomy of the ACL, ensuring optimal biomechanical function postsurgery.

#### 2.3.3. IDEAL reconstruction group (IDEAL group)

 For patients in the IDEAL group, femoral tunnel placement followed the IDEAL reconstruction technique as proposed by Pearle. The procedure used an anteromedial portal, positioning the femoral tunnel along the posterior cortical extension line and the resident’s ridge. The graft used in this group was a quadruple-strand semitendinosus tendon. The IDEAL technique emphasized several key principles: isometric positioning to maintain consistent graft length throughout knee motion, direct insertion at the ACL’s native insertion sites, eccentric placement within the anterior (high) and proximal (deep) regions of the ACL footprint, anatomical placement within the ACL footprint, and low tension to minimize stress on the graft during healing.

### 2.4. Postoperative rehabilitation

Both groups of patients followed the same postoperative rehabilitation protocol. During their hospital stay, patients received guidance from a physical therapist to perform isometric quadriceps exercises, passive closed-chain knee range-of-motion exercises, ankle pump exercises, and intermittent pneumatic compression therapy. Postoperatively, patients were allowed to begin partial weight-bearingimmediately,with the knee immobilized in full extension using a brace for 6 weeks. For the first 4 weeks postsurgery, knee flexion was limited to 100°. By 6 weeks, knee flexion was gradually increased to 120°, with further incremental increases thereafter. At 3 months postsurgery, patients were permitted to start jogging. By 9 months postsurgery, they were allowed to return to competitive sports, provided they had achieved adequate strength, stability, and functional recovery. This comprehensive rehabilitation plan aimed to ensure optimal recovery and functional outcomes for all patients.

### 2.5. Postoperative imaging and follow-up protocol

#### 2.5.1. Imaging confirmation

On the first day following surgery, patients underwent 3-dimensional computed tomography and MRI scans of the knee to confirm the position and orientation of the bone tunnels and to assess the condition of the graft. This imaging ensured the accuracy of the surgical procedure and the proper placement of the reconstruction.

#### 2.5.2. Discharge and initial follow-up

Patients were typically discharged between 2 and 4 days after surgery. A follow-up via telephone or an outpatient visit was conducted at 4 weeks postsurgery. During this follow-up, wound healing was observed, functional exercises were guided, and any complications such as surgical site infection, vascular or nerve injury, and knee stiffness were recorded.

#### 2.5.3. Long-term follow-up

Patients were scheduled for additional follow-up visits at 3, 6, and 12 months postsurgery. During these visits, knee function was assessed using the Lysholm Knee Scoring Scale, which evaluated pain, instability, and the ability to perform daily activities. In addition, the International Knee Documentation Committee (IKDC) Knee Examination Form was used to provide a standardized assessment of knee function, considering symptoms, sports activities, and overall knee functionality.

#### 2.5.4. Final follow-up and patient satisfaction

At the final follow-up, the Lachman test was performed to evaluate anterior knee stability and the integrity of the ACL graft. Patient satisfaction with the surgical outcome was assessed, with patients rating their satisfaction as very satisfied, satisfied, or dissatisfied.

### 2.6. Statistical analysis

Statistical analyses were performed using SPSS software (Version 27.0; IBM Corp., Armonk). Data were classified as either quantitative or categorical variables. For normally distributed quantitative data, intergroup comparisons were conducted using independent-samples *t* tests, with results expressed as mean ± standard deviation. Within-group comparisons across time points were evaluated using repeated-measures analysis of variance, followed by pairwise *t* tests where appropriate. Categorical variables were summarized as frequencies and percentages, and group differences were assessed using chi-square (χ^2^) tests. A two-tailed *P* < .05 was considered statistically significant.

## 3. Results

### 3.1. Demographic and preoperative characteristics

This study included a total of 76 patients, divided into the AR group (n = 39) and the IDEAL group (n = 37). Both groups were comparable in terms of demographic and preoperative characteristics. The average age was similar between the AR and IDEAL groups (32.5 ± 8.7 vs 33.0 ± 8.1 years, respectively), with no statistically significant difference (*P* > .05). The distribution of gender and the side of the affected knee were also balanced between the groups, with no significant differences observed (*P* > .05 for both). Preoperative duration, as measured in days from injury to surgery, showed a slight difference between the groups, with the AR group having a mean duration of 36.2 ± 21.8 days compared with 34.1 ± 19.6 days in the IDEAL group, although this difference was not statistically significant (*P* > .05). Preoperative functional scores, including the Lysholm and IKDC scores, were comparable between the groups, indicating similar baseline knee function (*P* > .05 for both). The number of patients with a positive Lachman test was equal in both groups (28 in each), confirming comparable levels of preoperative knee instability (Table 1). These findings indicate that the 2 groups were well-matched in terms of demographic and preoperative characteristics, providing a solid foundation for the comparative analysis of surgical outcomes.

**Table 1:** Preoperative and demographic characteristics of patients in the AR and IDEAL groups.

Characteristics	AR group (n = 39)	IDEAL group (n = 37)	*t* or χ^2^ value	*P* value
Age (yr)	32.5 ± 8.7	33.0 ± 8.1	*t* = .202	>.05
Gender (male/female)	23/16	20/17	χ^2^ = .131	>.05
Affected side (left/right)	16/23	17/20	χ^2^ = .091	>.05
Preoperative duration (d)	36.2 ± 21.8	34.1 ± 19.6	*t* = .891	>.05
Preoperative Lysholm score (points)	55.6 ± 7.2	54.9 ± 6.8	*t* = .356	>.05
Preoperative IKDC score (points)	54.2 ± 6.9	53.7 ± 6.2	*t* = .348	>.05
Preoperative Lachman test (positive cases)	28	28	χ^2^ = .068	>.05

AR = anatomical reconstruction, IDEAL = isometric, direct insertion, eccentrically located, anatomical, low tension reconstruction technique, IKDC = International Knee Documentation Committee, Lysholm = Lysholm Knee Scoring Scale.

### 3.2. Lysholm scores at different postoperative time points

Follow-up data were available for all 76 patients at 3, 12, and 24 months postsurgery. The Lysholm scores, which assess knee function, demonstrated significant improvements over time in both the AR and IDEAL groups. At 3 months postsurgery, the Lysholm scores were comparable between the 2 groups, with the AR group scoring 65.7 ± 9.5 and the IDEAL group scoring 64.3 ± 8.8, showing no statistically significant difference (*P* = .814). By 12 months postsurgery, both groups showed further improvement in Lysholm scores, with the AR group scoring 74.1 ± 8.0 and the IDEAL group scoring 75.6 ± 7.7, but no significant difference was observed between the groups (*P* = .623). At the time of follow-up, both groups achieved substantial increases in Lysholm scores, with the AR group reaching 87.9 ± 9.3 and the IDEAL group 88.4 ± 8.2. Despite these improvements, the differences between the groups remained statistically insignificant (*P* = .707). The *F* values for both groups indicated a significant improvement over time (*P* < .01), reflecting the overall efficacy of both reconstruction techniques in enhancing knee function (Table 2). However, no significant differences were found between the AR and IDEAL groups at any of the assessed time points, suggesting that both surgical methods are equally effective in improving knee function post-ACL reconstruction.

**Table 2:** Comparison of Lysholm scores between AR and IDEAL groups at different postoperative time points.

Group	3 mo postsurgery	12 mo postsurgery	24 mo postsurgery	*F* value	*P* value
AR group	65.7 ± 9.5*	74.1 ± 8.0*,†	87.9 ± 9.3*,†,‡	31.02	<.01
IDEAL group	64.3 ± 8.8*	75.6 ± 7.7*,†	88.4 ± 8.2*,†,‡	43.23	<.01
*t* value	.236	.492	.378		
*P* value	.814	.623	.707		

AR = anatomical reconstruction, IDEAL = isometric, direct insertion, eccentrically located, anatomical, low tension reconstruction technique, Lysholm = Lysholm Knee Scoring Scale.

* Compared with preoperative values, *P* < .01.

† Compared with 3 months postoperative, *P* < .01.

‡ Compared with 12 months postoperative, *P* < .01.

### 3.3. IKDC scores at different postoperative time points

Follow-up data were available for all 76 patients at each assessment. The IKDC scores, used to evaluate knee function, showed marked improvement in both the AR and IDEAL groups over the course of the study. At 3 months postsurgery, the IKDC scores were similar between the groups, with the AR group scoring 66.8 ± 10.5 and the IDEAL group scoring 64.9 ± 9.6, indicating no statistically significant difference (*P* = .920). By 12 months postsurgery, both groups exhibited further enhancement in their IKDC scores, with the AR group reaching 75.9 ± 8.6 and the IDEAL group 72.9 ± 8.9. Although the IDEAL group showed slightly lower scores compared with the AR group, this difference was not statistically significant (*P* = .606). At the 24-month follow-up, both groups demonstrated substantial improvements, with the AR group achieving 91.4 ± 7.5 and the IDEAL group 87.6 ± 7.2. Despite the higher scores observed in the AR group, the differences between the 2 groups were not statistically significant (*P* = .346). The *F* values indicated significant within-group improvements over time (*P* < .01) for both groups, underscoring the effectiveness of both surgical techniques in improving knee function post-ACL reconstruction (Table 3). The results suggest that while both AR and IDEAL reconstructions are effective in restoring knee function, there are no significant differences in the overall outcomes between the 2 approaches.

**Table 3:** Comparison of IKDC scores between AR and IDEAL groups at different postoperative time points.

Group	3 mo postsurgery	12 mo postsurgery	24 mo postsurgery	*F* value	*P* value
AR group	66.8 ± 10.5*	75.9 ± 8.6*^,^†	91.4 ± 7.5*,†,‡	48.49	<.01
IDEAL group	64.9 ± 9.6*	72.9 ± 8.9*,†	87.6 ± 7.2*,†,‡	47.69	<.01
*t* value	.102	.518	.950		
*P* value	.920	.606	.346		

AR = anatomical reconstruction, IDEAL = isometric, direct insertion, eccentrically located, anatomical, low tension reconstruction technique, IKDC = International Knee Documentation Committee.

* Compared with preoperative values, *P* < .01.

† Compared with 3 months postoperative, *P* < .01.

‡ Compared with 12 months postoperative, *P* < .01.

## 4. Discussion

ACL tears are among the most common and debilitating knee injuries, particularly in athletes and individuals engaged in high-demand physical activities. Effective surgical reconstruction is critical for restoring knee stability and function, which are essential for returning to pre-injury activity levels. Two widely adopted ACL reconstruction techniques are AR and IDEAL reconstruction.^[18–20]^ AR aims to replicate the native ACL anatomy by precisely positioning the graft at its original femoral and tibial insertion sites, thereby restoring physiological knee biomechanics and potentially improving long-term outcomes. In contrast, IDEAL reconstruction incorporates specific principles – including isometric graft placement, eccentric tunnel positioning, and low tension – to optimize graft function and reduce the risk of failure.^[21,22]^ This study evaluated the impact of AR versus IDEAL reconstruction on postoperative functional recovery in patients with ACL tears. Primary outcomes included the Lysholm Knee Scoring Scale and the IKDC score, both of which are well-validated tools for assessing knee function and overall patient-reported outcomes following ACL reconstruction.^[23,24]^ In this cohort, both AR and IDEAL groups demonstrated significant improvements in knee function over time, with no statistically significant differences between techniques at any measured time point. These findings indicate that, within the limitations of this study, AR and IDEAL reconstruction yielded comparable short- to mid-term functional outcomes following ACL reconstruction.

One possible explanation for the comparable outcomes between AR and IDEAL reconstructions lies in the fundamental principles underpinning each technique. AR focuses on restoring the ACL to its native anatomical footprint, which is thought to more accurately replicate the physiological biomechanics of the knee. This method has been associated with improved rotational stability and restoration of natural knee kinematics postoperatively. By positioning the graft precisely within the native ACL footprint, AR aims to closely mimic the original ligament’s function, potentially enhancing long-term outcomes and reducing the risk of reinjury.^[25,26]^ In contrast, the IDEAL reconstruction technique incorporates a set of guiding principles – isometric graft placement, direct insertion at the native insertion sites, eccentric tunnel positioning, anatomical placement within the ACL footprint, and low graft tension. These principles are designed to optimize graft biomechanics, minimize elongation, and reduce the risk of failure. In particular, isometric positioning ensures that graft length remains consistent throughout the full range of motion, which may contribute to enhanced stability and functional performance during dynamic activities.^[27,28]^ Furthermore, maintaining low tension during fixation helps to reduce mechanical stress on the graft during the healing process, potentially promoting superior biological integration and long-term durability of the reconstructed ligament.

Despite the theoretical advantages of each technique, the absence of statistically significant differences in functional outcomes between the AR and IDEAL groups in this study suggests that both methods may be similarly effective in achieving the primary objective of ACL reconstruction – restoring knee stability and function. Several factors may account for these comparable results. First, both techniques emphasize precise graft placement within the anatomical ACL footprint, a critical determinant of postoperative success. Whether performed via the anatomical approach or the IDEAL method, accurate tunnel positioning is likely pivotal in achieving favorable clinical outcomes.^[29,30]^ Second, both groups followed an identical standardized postoperative rehabilitation protocol, which may have further contributed to the similarity in results. This structured program, incorporating progressive weight-bearing, range-of-motion exercises, and targeted strength training, is fundamental to optimizing functional recovery, irrespective of the surgical technique employed.^[31,32]^

Both anatomical and IDEAL reconstruction techniques aim to restore knee stability by optimizing graft placement and tensioning. However, graft fixation methods play a crucial role in postoperative functional recovery by influencing graft stability, integration, and biomechanical performance. In our study, the IDEAL reconstruction group utilized a quadruple-strand semitendinosus tendon fixed within the femoral tunnel positioned along the posterior cortical extension line and the resident’s ridge, emphasizing low-tension graft placement to minimize stress and enhance healing. Existing literature suggests that suspensory fixation systems such as the Endobutton provide excellent initial stability but may allow micromotion within the tunnel, potentially leading to tunnel widening over time.^[33,34]^ Interference screws (bioabsorbable or metallic) offer rigid fixation and closer contact between the graft and bone tunnel, potentially promoting earlier graft incorporation.^[35]^ Concerns have been raised regarding the use of interference screws with soft tissue grafts, including potential graft damage during insertion and reduced fixation strength. However, biomechanical studies have not demonstrated direct graft cutting by the screw, regardless of its positioning. Proper placement of the interference screw, particularly in a central position, may enhance bone-tendon contact and mitigate these risks.^[36]^ Although our study did not stratify outcomes based on specific fixation devices, future research should explore whether differences in fixation methods contribute to variations in functional recovery. A comparative analysis of fixation techniques in the context of anatomical versus IDEAL reconstruction could provide deeper insights into their biomechanical and clinical implications.

The findings of this study have relevant clinical implications. Given that both AR and IDEAL reconstructions yielded comparable functional outcomes, surgeons may have the flexibility to select the technique based on patient-specific factors, surgical expertise, and individual clinical circumstances. For example, in cases with substantial damage to the ACL footprint, the IDEAL technique may be advantageous due to its emphasis on isometric positioning and low graft tension. Conversely, in patients with relatively preserved ACL remnants, AR may be preferable for restoring the knee’s native biomechanics.^[37,38]^ Furthermore, the absence of significant differences in outcomes between the 2 approaches supports the notion that both are viable options for ACL reconstruction, thereby providing surgeons with multiple strategies to achieve satisfactory results. This is particularly pertinent in clinical practice, where the choice of surgical technique may be influenced by the surgeon’s familiarity with the procedure, available instrumentation, and patient preferences.^[39,40]^

The limitations of this study need to be pointed out. This study was retrospective in nature, which may introduce selection bias. In addition, another important limitation of this study is the relatively small sample size and the absence of an a priori statistical power analysis. Although no statistically significant differences were observed between the AR and IDEAL groups, this study may have been underpowered to detect small or moderate clinically meaningful differences, thereby increasing the possibility of a type II error. Consequently, the absence of significant differences should not be interpreted as definitive evidence of equivalence between the 2 techniques. Larger prospective multicenter studies with predefined sample size calculations are required to provide more robust statistical evidence. Future research should include prospective, randomized trials with larger cohorts and formal power calculations to validate these findings and evaluate the long-term durability of each technique. Furthermore, it would be valuable to investigate the impact of these reconstruction techniques on other important outcomes, such as the rate of return to sport, patient-reported quality of life, and the incidence of post-traumatic osteoarthritis. Understanding these factors will provide a more comprehensive assessment of the benefits and limitations of AR and IDEAL reconstruction techniques, ultimately guiding clinical decision-making and improving patient care. Although all procedures were performed by experienced surgeons within the same surgical team, the retrospective nature of this study means that surgeon-related variability and subtle differences in surgical decision-making cannot be completely excluded. In addition, potential learning-curve effects associated with technical nuances of the IDEAL reconstruction technique may still have influenced outcomes.

In addition, the postoperative evaluation indicators used in this study were relatively limited and primarily based on patient-reportedoutcome measures, including the Lysholm and IKDC scores. Although these instruments are widely validated and clinically meaningful, they may not fully capture objective biomechanical knee stability. This study lacked quantitative stability assessments such as KT-1000/KT-2000 arthrometer testing, pivot-shift grading, stress radiography, or dynamic imaging evaluations. Therefore, subtle differences in graft function and rotational stability between AR and IDEAL reconstruction may not have been adequately detected.

## 5. Conclusions

In conclusion, both AR and IDEAL reconstruction techniques are effective in restoring knee function following ACL reconstruction, with no significant differences in postoperative functional outcomes as measured using Lysholm and IKDC scores. These findings suggest that surgeons can achieve favorable results with either technique, depending on the specific clinical context and patient characteristics. Larger, adequately powered prospective randomized studies are warranted to determine whether meaningful differences exist and to assess long-term safety and durability.

## Acknowledgments

The authors thank everyone involved in this study for their efforts.

## Author contributions

**Conceptualization:** Jianbin Gu, Xiaoqi Zhou, Yanpeng Fu, Xun Zhen, Zhizhu Xu, Zhaoyu Gong.

**Data curation:** Jianbin Gu, Xiaoqi Zhou, Yanpeng Fu, Xun Zhen, Zhizhu Xu, Zhaoyu Gong.

**Formal analysis:** Jianbin Gu, Xiaoqi Zhou, Yanpeng Fu, Xun Zhen, Zhizhu Xu, Zhaoyu Gong.

**Funding acquisition:** Jianbin Gu, Xiaoqi Zhou, Yanpeng Fu, Zhaoyu Gong.

**Investigation:** Jianbin Gu, Yanpeng Fu.

**Writing – original draft:** Jianbin Gu.

**Writing – review & editing:** Jianbin Gu.
